# Regulation of HMGB1 Release in Health and Diseases

**DOI:** 10.3390/cells12010046

**Published:** 2022-12-22

**Authors:** Haichao Wang

**Affiliations:** The Feinstein Institutes for Medical Research, 350 Community Drive, Manhasset, NY 11030, USA; hwang@northwell.edu

Almost a half century ago, a group of nuclear proteins were co-purified with histones from calf thymus and termed as “high mobility group” (HMG) proteins because of their relative rapid mobility on SDS-PAGE gels [1]. These HMG proteins are subsequently divided into three groups based on their size, sequence similarities and DNA binding properties: (i) the HMGB1/2 family; (ii) the HMGN1/2 family; and (iii) the HMGA1 family [2]. Among them, HMGB1 is the most ubiquitous, abundant, and evolutionarily conserved protein in eukaryotes, exhibiting 99% amino acid sequence homology between rodents and humans. As a non-histone chromosomal protein, nuclear HMGB1 has been implicated in diverse cellular functions, including determination of nucleosomal structure and stability, and binding of transcription factors to their cognate DNA sequences [3]. Since the seminal discovery of extracellular HMGB1 as a proinflammatory mediator of lethal systemic inflammation [4], there has been explosive interests in intensive research about mechanisms underlying the regulation of HMGB1 release and action, as well as its extracellular roles in various inflammatory diseases. In this Special Issue, we have provided comprehensive reviews and an example of on-going research about the divergent mechanisms underlying the regulation of HMGB1 release and action by exogenous and endogenous molecules, as well as its pathogenic roles in various infection- and injury-elicited inflammatory conditions.

In the first chapter [5], Ge et al. provided a comprehensive review of the regulatory mechanisms of HMGB1 release by various immune cells under different inflammatory conditions. This chapter begins with a brief introduction of the initial discovery of HMGB1 first as a ubiquitous nuclear protein, and then as a damage-associated molecular pattern molecule (DAMPs) capable of binding various patter recognition receptors (PRRs) such as the Toll-Like Receptor 4 (TLR4) and the Receptor for Advanced Glycation End product (RAGE). Subsequently, the authors have discussed the impact of extracellular HMGB1 on immune functions of neutrophils, macrophages/monocytes, dendritic cells, and T lymphocytes. Finally, these authors have summarized the important role of extracellular HMGB1 in the pathogenesis of various inflammatory diseases such as sepsis, autoimmune diseases, lung inflammatory diseases, cardiac and hepatic injury, and encephalopathy.

In the second chapter [6], Zhu et al. have specifically presented an updated summary of mechanisms underlying endogenous regulation and pharmacological modulation of bacterial infection (sepsis)-induced HMGB1 release and action. This chapter begins with a brief comparison of the distinct mechanisms underlying the regulation of early cytokines (e.g., TNF) by cell-surface PRRs (e.g., TLR4) and late-acting mediators (i.e., HMGB1) by cytoplasmic PRRs (e.g., caspase-11/4/5). It then continues with a discussion of the pathogenic role of extracellular HMGB1 in the regulation of dysregulated inflammation and immunosuppression in conjunction with different PRRs (e.g., TLR4 and RAGE). Next, the authors have discussed several positive regulators (e.g., type I and type II IFNs and serum amyloid A, SAA) that can trigger HMGB1 release by inducing NLRP3-dependent inflammasome activation and pyroptosis, as well as several negative regulators of HMGB1 release that include cationic anti-inflammatory molecules (e.g., spermine), and vagus nerve nicotinic cholinergic signaling pathway. Finally, these authors have listed several pharmacologic agents capable of regulating HMGB1 release and action such as small molecule herbal components as well as endogenous proteins (such as tetranectin) that could affect HMGB1 release and/or action to prevent dysregulated inflammatory responses to lethal infections (such as COVID-19).

In the third chapter [7], Watanabe et al. have specifically discussed the role of HMGB1-RAGE axis in the regulation of immune tolerance in the context of systemic lupus erythematosus and vasculitis. This chapter begins with a brief introduction of the role of immune tolerance in autoimmunity and autoimmune diseases, and then continues with a discussion of the mechanisms underlying the regulation of HMGB1 release, as well as its extracellular functions in propagating inflammatory responses through RAGE-dependent signaling pathways. Furthermore, the authors have discussed the important role of the HMGB1-RAGE axis in systemic lupus erythematosus such as by providing a functional platform for cross-talking with other cytoplasmic receptors such as TLR9. Finally, the authors have summarized the role of HMGB1-RAGE axis in the regulation of other diseases (such as autoimmune vasculitis and ischemic injury) and immune tolerance through interacting with other endogenous proteins (such as haptoglobin and complement 1Q, C1Q).

In the fourth chapter [8], Foglio et al. have specifically summarized the extracellular role of HMGB1 in the inflammatory response to myocardial injuries. First, the authors have introduced the regulatory role of immune response in myocardial injury-elicited inflammatory responses, and then presented some evidence to support extracellular HMGB1 as a DAMP that amplifies inflammatory responses via various PRRs (such as CXCR4, RAGE, and TLR4). Moreover, the authors have discussed the role of extracellular HMGB1 in the regulation of tissue inflammatory injury and repair that are mediated by various types of innate immune cells such as neutrophils, macrophages/monocytes, dendritic cells, as well as components of the adaptive immune system such as T and B lymphocytes. Finally, the authors have discussed potential mechanisms by which HMGB1 mediates cardiac repair and regeneration after myocardial ischemia/reperfusion injuries.

In the fifth chapter [9], Yang et al., have provided new evidence to support neurons as a source of HMGB1 release and a molecular driver of nerve injury-associated inflammation. First, the authors have introduced the possible role of nociceptive neurons in activating inflammatory responses by releasing a variety of vasoactive and immune stimulating molecules, such as histamine, neuropeptides, and HMGB1. Then, these authors have proposed a pathogenic role HMGB1 in various neuroinflammatory diseases via TLR4-dependent mechanisms upon its active release by nociceptive neurons. Finally, the authors have discussed several pharmacological strategies to prevent neuroinflammatory responses including HMGB1-neutralizing antibodies and other antagonists. The discovery of neuronal HMGB1 as a major driver of neuroinflammation support the dual role of the nervous system in positive and negative regulation of inflammatory responses to infections and injuries.

In the sixth chapter [10], Saxena et al. have specifically discussed the potential role of extracellular HMGB1 in perioperative neurocognitive disorders. First, the authors have briefly introduced the nomenclature and structure of HMGB protein family, its post-translational modifications, as well as mechanisms underlying its extracellular secretion or passive release. Then the authors have summarized the role of nuclear and extracellular HMGB1 in the regulation of gene expression and inflammatory responses, respectively. Next, the authors have continued with a discussion of the molecular signaling involved in the regulation of HMGB1-mediated inflammatory responses, as well as anti-inflammatory signaling leading to immunosuppression. Afterward, the authors have introduced the concept of perioperative neurocognitive disorders as well as its inflammatory pathogenesis including the contribution of HMGB1-induced inflammation to traumatic injury-elicited cognitive decline. Finally, the authors have discussed possible therapeutic strategies for attenuating HMGB1-induced neuroinflammation such as neutralizing antibodies, peptide antagonists (e.g., the B box of HMGB1), as well as small molecule inhibitors (such as glycyrrhizin, Polyunsaturated Fatty Acid and flavonoids).

In the last chapter [11], Shih et al. examined the pathogenic role of cochlear HMGB1 in a murine model of noise-induced hearing loss by pharmacologically manipulating cochlear HMGB1 level or activities using recombinant protein or neutralizing antibodies. The authors demonstrated that recombinant HMGB1 activated primary cochlear cells to up-regulated the inducible nitric oxide synthase (iNOS) in vitro, and noise exposure increased cochlear HMGB1 expression and oxidative stress in vivo. Moreover, pharmacological administration of HMGB1-neutralizing antibodies diminished the noise-induced production of reactive oxygen species in the cochlea, which was associated with a reduction of cochlear hair cell loss. It provided some evidence to support a potentially pathogenic role of cochlear HMGB1 in noise-induced hearing loss.

In summary, this special issue has provided comprehensive review of the intricate molecular mechanisms underlying the regulation of HMGB1 release and action by various PRRs as well as various endogenous molecules and exogenous pharmacological agents. It also contains thorough discussions of the pathogenic roles of extracellular HMGB1 in various inflammatory diseases such as sepsis, immune tolerance, ischemia/reperfusion injury, neuropathological pain, and perioperative neurocognitive disorders. Last but not the least, it provides an example of on-going investigations of the pathogenic role of HMGB1 in various inflammatory diseases. Therefore, this Special Issue will serve as an excellent reference for beginners to gain an comprehensive understanding of extensive HMGB1 research during the last 25 years.
